# Machining performance investigation on 17-4PH steel material with innovative textured tools

**DOI:** 10.1038/s41598-026-42889-y

**Published:** 2026-03-12

**Authors:** P. Sivaiah, Karthik M C Rao, C. Yuvaraj, P. Mallikarjuna, V. Chengal Reddy, T. Nishkala

**Affiliations:** 1 Department of Mechanical Engineering, Madanapalle Institute of Technology & Science (MITS), Deemed to be University, Madanapalle, Andhra Pradesh 517325 India; 2 Manipal Institute of Technology, Manipal Academy of Higher Education, Manipal, India; 3 Department of Mechanical Engineering, Annamacharya Institute of Technology & Sciences, Kadapa, Andhra Pradesh 516 003 India; 4 Department of Mechanical Engineering, Annamacharya Institute of Technology & Sciences, Tirupati, Andhra Pradesh, 517520 India

**Keywords:** Machining, Hybrid textured tools, 17-4PH steel, Turning, Engineering, Materials science

## Abstract

The machinability characteristics during turning process are noticeably influenced by the texture design particularly single pattern or dual pattern about surface textured tools. In this context, novel dual pattern textured tools were developed in the present work and compared its machinability performance with conventional tools respectively in tuning of 17-4PH steel under wet cooling conditions. Dual pattern textured tools consists of continuous microgrooves which are combination of parallel and perpendicular to the chip flow direction and also, have microgrooves parallel to principal cutting edge and secondary cutting-edge to enhance. In this work, investigation was done at different cutting velocity. Cutting temperature (T), surface roughness (R_a_), and tool flank wear (V_b_) were shown to be significantly decreased by hybrid textured tools compared to conventional tools, with maximum values of 26%, 28%, and 21%, respectively. Further, developed textured tools found with low adhesion and abrasion wear when compared to conventional tool. Moreover, interaction effect of input process variables on outputs was studied via interaction plots. From results it was retrieved that hybrid textured tools served as coolant storages sites to supply coolant to the cutting zone effectively.

## Introduction

Performance of any machining operation significantly affected by the magnitude of cutting temperature. Therefore, cutting temperature control is essential during machining operation. Nevertheless, flood cooling is not an environmentally friendly method and fails at higher levels in the machining zone^1^. In this connection, targeted task in this work is to maximize the coolant supply to the cutting zone. In the literature, researchers identified advancements in the machining techniques to improve the productivity of different processes^2^. Among all, textured tools technique is the one which involves no other cooling technique requirement to improve the productivity. Still, textured tools could allow coolant storage in the texture geometry and maximizes coolant delivery to the cutting zone by facilitating continuous coolant supply to the tool-chip contact under various cooling strategies. Recently, few studies were reported on the machinability studies using different textured tools with different surface texture geometry under various cutting environments. Sun et al.^3^ assessed the dry turning operation results while machining of AISI 1045 material using hybrid, single pattern textured tools and conventional tools. It was explained that positive results in hybrid textured tools results in effective coolant supply leads to low friction and high shear angle. Singh et al.^4^ studied the impact of single pattern textured in dry, MQL, Nano-MQL condition. They pragmatic that low surface roughness and low tool wear in nano-MQL integrated with textured tool owing to low friction over other cutting conditions. Hao et al.^5^ investigated Ti6Al4V material machinability with single and hybrid textured design under different cutting conditions. According to reports, textured tool under MQL conditions performed better due to a thick lubricating film at the cutting zone. They concluded that microgrooves have coolant guiding effect. Zheng et al.^6^ examined the impact of various single pattern textured tools on the dry turning of titanium material. According to reports, textured tools have lower cutting forces and BUE than conventional tools because of their smooth cutting movements. Additionally, it was determined that the placement of the texture had a significant impact on the performance of textured tools. Kumar and Raj^7^ examined the machinability characteristics of turning operation with textured tools while dry cutting of AISI 4340 material. Results show that chip entrapment was discovered in textured tools, which results in low tool-chip contact length (L) and better results. Siju el al.^8^ created dual textured tools and used them to conduct research on dry turning titanium alloy. They noticed low cutting forces in the hybrid tools. Further, noticed less friction in hybrid textured tool. Additionally, it was practical for all tools to use adhesive wear as a tool wear mechanism. Sencan et al.^9^ used SiO₂ as nanoparticles in MQL coolant and investigated the turning performance indices under nano-MQL with different percentages, MQL and dry conditions respectively in cutting of AISI304 materials. They found better lubrication at the cutting zone causes improved results at 0.5% nano-MQL cooling over other conditions. Zhou et al.^10^ performed dry turning on AISI 304 material found low BUE and low cutting force in single pattern textured tools owing to tool-chip contact length. Roushan and Chetan^11^ different discrete, continuous and hybrid texture design tools were fabricated and investigated its machinability while turning of PH 13 − 8 Mo steel material. Because hybrid textured tools reduce friction at the cutting zone, they observed the greatest turning performance. Further, concluded that derivative cutting mechanism during cutting operation greatly influenced by the geometry and position of texture. Bharath and Venkatesan^12^ different single pattern textured tools machinability was evaluated while turning of Inconel 713 C material under solid lubrication condition. They noted considerable improvement of turning process with honeycomb textured tool due to rise in shear angle. Further, found less adhesion and abrasion wear in honeycomb textured tool with solid lubricant then other conditions respectively. recently, several single and dual textured tools were tested when cutting challenging materials in varied cooling settings and it was shown that textured tools increased machinability^13–20^. 17-4PH steel is having poor machinability index under conventional cooling condition or dry condition due to exceptional mechanical properties^21^. Since, this material has applications in the different emerging field, the efforts should be made to rise the machinability index for productivity improvement in processing of this material. Few studies in the literature have attempted to examine the machinability of this material under various cutting circumstances. Mohanty et al.^22^ evaluated the PVD coated and untreated tools machinability performance while dry turning of 17 − 4 PH. They found high cutting temperature in coated tools compared to uncoated tools. However, considerable enhancement in tool life and chip morphology was found in coated tools over uncoated tools due to anti friction properties of coated tools. Khani et al.^23^ noticed substantial machinability improvement with emerging cryogenic cooling technique compared to conventional cutting condition. Further, it was reported that hybrid machining technique combination of cryogenic and hot machining significantly improved the machinability results. Investigations showed that found best results with cryogenic and MQL conditions while turning 17 − 4 PH material due low cutting temperatures^23–25^. Sivaiah et al.^26^ reported that textured tools provide site to store coolant under wet cooling and found improved turning process performance in textured tools in turning of 17-4PH steel. Shi et al. Laser-processed rake face textures were applied to carbide tools to enhance the machining of nickel-based superalloys. Under mixed nanofluid MQL, textured tools exhibited superior machinability, confirming that optimized tool textures in combination with MQL improve both machining efficiency and tool life^27^. Tian et al.^28^ studied dry cutting of quenched AISI5140 steel using combined coating and textured tool. They noticed paramount machinability performance. Further concluded that the proposed approach also minimizes cutting fluid use and energy consumption, offering environmentally friendly machining benefits. George et al.^29^ found that channel-textured tools enhanced the dry machining of KhN67VMTYu, extended the tool life by 58% and lower ‘Ra’ by 49% respectively. Improved heat dissipation and reduced contact stress mitigated wear, resulting in superior performance compared to conventional tools. de Souza et al.^30^ noticed that textured PCD tools particularly with perpendicular textures combined with MoS₂, enhanced AA2011-T4 turning by lowered ‘Ra’ 49%, reduced aluminum adhesion, and facilitating chip removal, resulting in superior tool performance. Fareedh et al.^31^ found that micro-dimpled cutting tools with a low shape factor (SF = 0.4) effectively reduce chip adhesion and built-up edge during Ti-6Al-4 V turning under MQL. Relative to non-textured tools. Jiang et al.^32^ different texture geometry parameters were considered and optimized the texture design using Response Surface Methodology (RSM) to obtain the paramount performance of dry turning process while machining gray cast iron. Further, experimental results validated the accuracy and practical effectiveness of the optimized configuration. Divya et al.^33^ filled WS₂ solid lubricant in the developed textured tools and found improved machinability during turning of Inconel 718 due to markedly decreased friction at the cutting zone. Further, optimum process parameters were identified by using Taguchi approach. Yao et al.^34^ investigated the influence of tool and texture parameters on subsurface damage during CFRP machining and modelled damage behaviour through RSM. Further, found 50% reduction in subsurface damage with optimized textured tools relative to conventional tools using hybrid metaheuristic optimization approach. Mia et al.^35^ studied the sustainable end milling of hardened AISI 4140 steel under MQL, using response surface methodology to model specific cutting energy and surface roughness. Key process parameters and their interactions were identified through ANOVA and comprehensive statistical analyses, and optimization using RSM and Taguchi approaches provided consistent and reliable predictions of optimal machining conditions. Likewise, authors used optimization approaches and statistical tools for optimization and analyses of various processes^36–40^.

The literature study revealed that machinability of any material during turning operation is significantly depends on the geometry of surface texture, design of surface texture and cutting environment. So, in this work, carried out machinability evaluation with novel hybrid textured tools during turning of 17-4PH material under wet cooling environment. An innovative hybrid surface texture design consists of combination of continuous parallel and perpendicular microgrooves against chip flow direction and also, have microgrooves parallel to principal cutting edge and secondary cutting-edge to enhance. The main intension to select the considered surface textured design is to deliver coolant incessantly. According to the literature, no machinability data on turning 17-4PH material with such developed tools is available. The current work’s results were compared to those obtained with untextured tools.

## Materials and methods

**Fig. 1:**
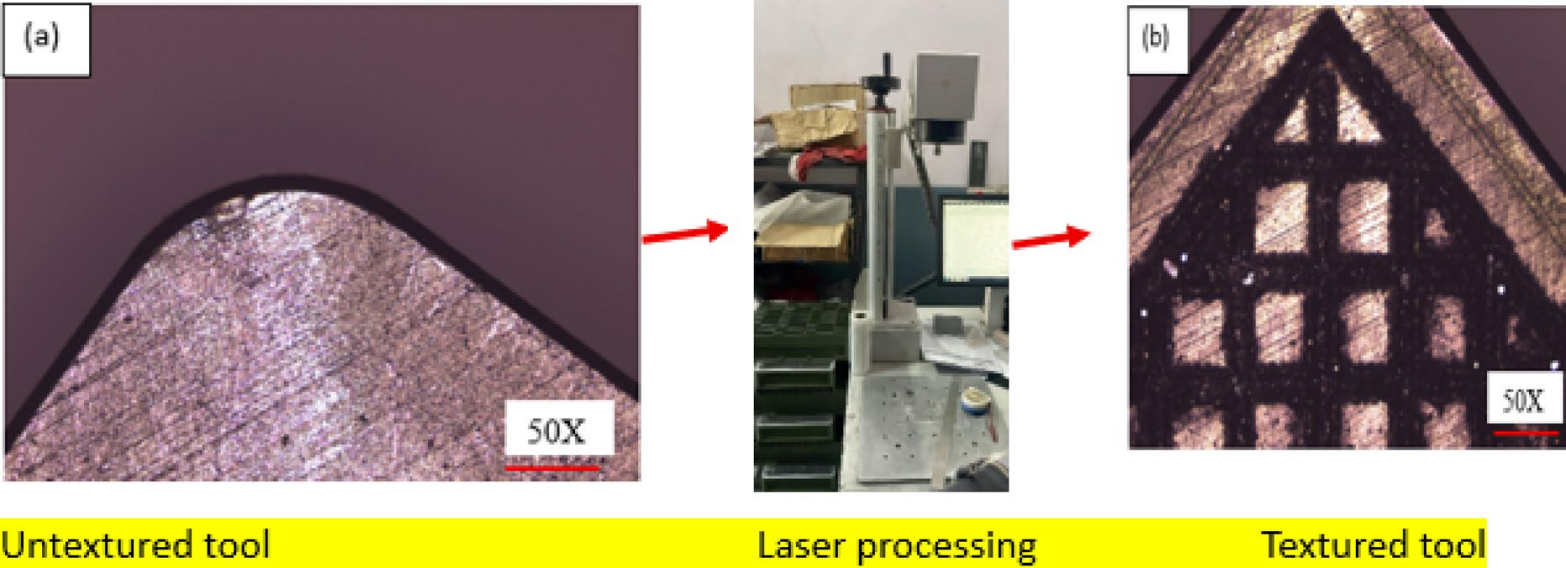
Developed hybrid surface textured tool (a)Untextured tool (b) Textured tool.

**Fig. 2:**
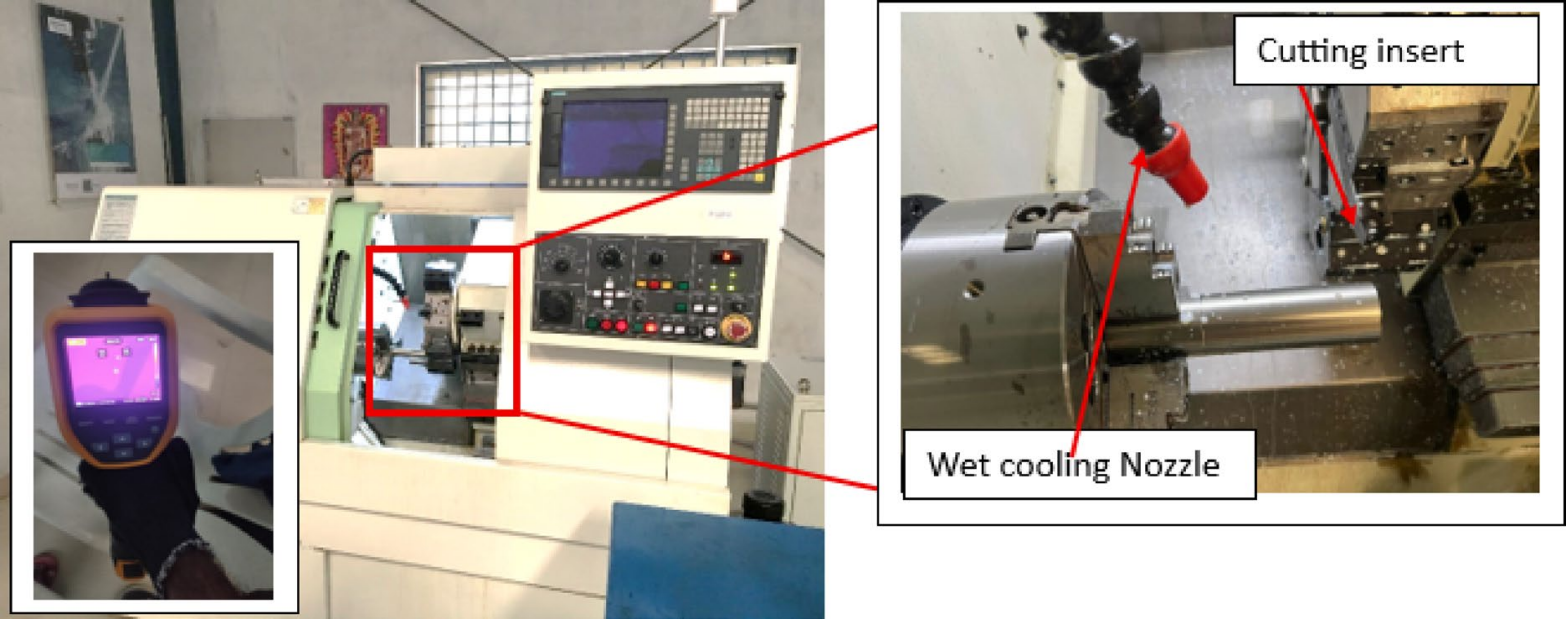
Machine with tool-work machining zone.

**Fig. 3:**
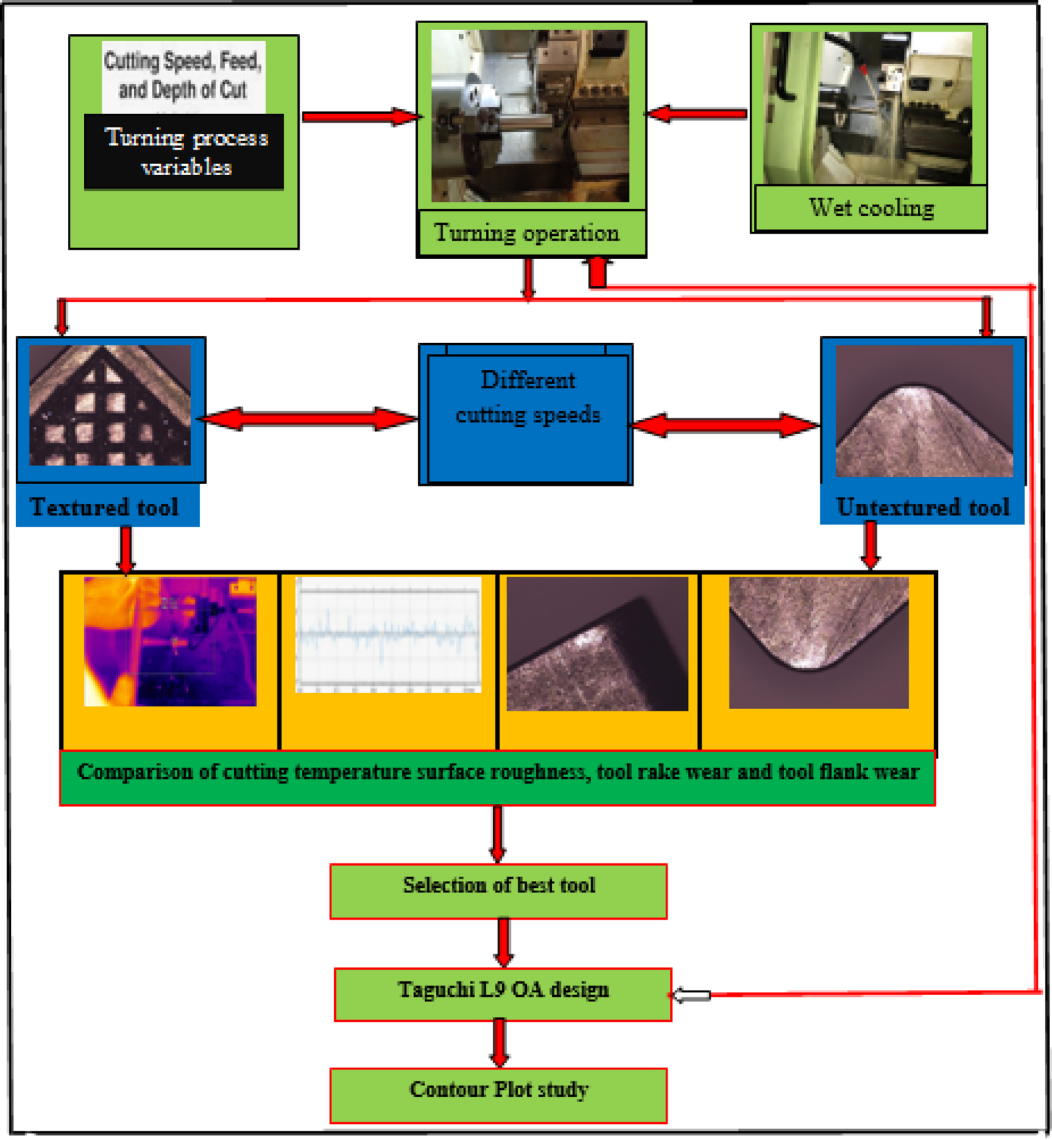
Methodology.

The developed novel textured tool using fibre laser processing technique is depicted in Fig. 1. The innovation lies in textured tool is that textured microgrooves considered in this study are combination of parallel and perpendicular to the against chip flow direction, also, have microgrooves parallel to principal cutting edge and secondary cutting-edge to enhance the coolant supply to the cutting zone. The horizontal and vertical distance between two consecutive microgrooves is kept at 100 μm and depth of the grooves 100 μm. These developed tools and untextured tools were used to machine 17-4PH material to evaluate the turning process machinability characteristics under wet cooling condition. The workpiece (⌀26 mm × 150 mm) was tested in triplicate, and the average values were reported. Cutting inserts were mounted using a PSBNL 2020 K12 tool holder (Taegu Tech). The machining zone image under wet cooling is shown in Fig. 2. During machining operation, each run was conducted for 200 mm machining length with fresh cutting tip. During experimentation, a 10 mm external nozzle was used and supplied coolant with flow rate of 5 L/hr^41^. As per the tool manufacturer advice, the present study utilized tungsten carbide (SNMA120408) tools to machine the chosen material. Machinability was assessed in the current work at cutting velocity (v) values of 80 and 113 m/min while maintaining constant feed rate (f) of 0.1 mm/rev and depth of cut (a_p_) of 0.1 mm. Additionally, nine experiments were performed using textured tools based on the Taguchi L_9_ orthogonal array to investigate the interaction effects of various process parameters on the responses, with the results presented in Table 1. The cutting parameter levels were selected based on the pilot experiments. One of the sensitive parameters in turning operation is cutting velocity which directly alters the thermo-mechanical conditions at the tool–workpiece interface in turning operation. Even slight changes in cutting velocity can cause significant variations in the tool–chip contact temperature The recommended ‘v’ values were selected as per trail runs. The flank wear (V_b_) values and rake wear (K_t_) was measured using Zeiss make AXIOLAB A1 model optical microscope. ISO 3685:1993 standard followed to measure it. SJ-310 model Talysurf tester was used to measure the ‘R_a_’ of machined workpiece. The ‘T’ was recorded using Fluke make Model TiS55 + thermal image camera. An emissivity value of 0.35 was chosen and this value was selected based on commonly reported emissivity ranges for stainless steels in machining studies. The cutting temperature was measured during machining at the beginning, midpoint, and end of the cut, and the average of these readings was taken as the final value. The thermal camera was calibrated against a blackbody reference over the anticipated temperature range. The emissivity was set according to the workpiece material, and the system was allowed to stabilize prior to measurements, ensuring accurate and reliable temperature data. Methodology followed in the present work is depicted in Fig. 3.

**Table 1:** Experimental results.

S.No.	‘v’ (m/min)	‘f’ (mm/rev)	‘a_*p*_’ (mm)	‘T’(^o^C)	‘*R*_a_’(µm)	‘V_b_’(µm)	‘K_t_’(µm)
1	80	0.05	0.1	55	1.4	180	237
2	80	0.075	0.2	64	1.48	214	282
3	80	0.1	0.3	73	1.76	271	357
4	96	0.05	0.2	75	1.22	190	250
5	96	0.075	0.3	79	0.95	274	361
6	96	0.1	0.1	85	1.38	281	370
7	113	0.05	0.3	95	0.91	290	382
8	113	0.075	0.1	105	1.25	305	402
9	113	0.1	0.2	120	1.18	320	421

## Results and discussion

### Wet cooling and hybrid texture tool impact on ‘T’

**Fig. 4:**
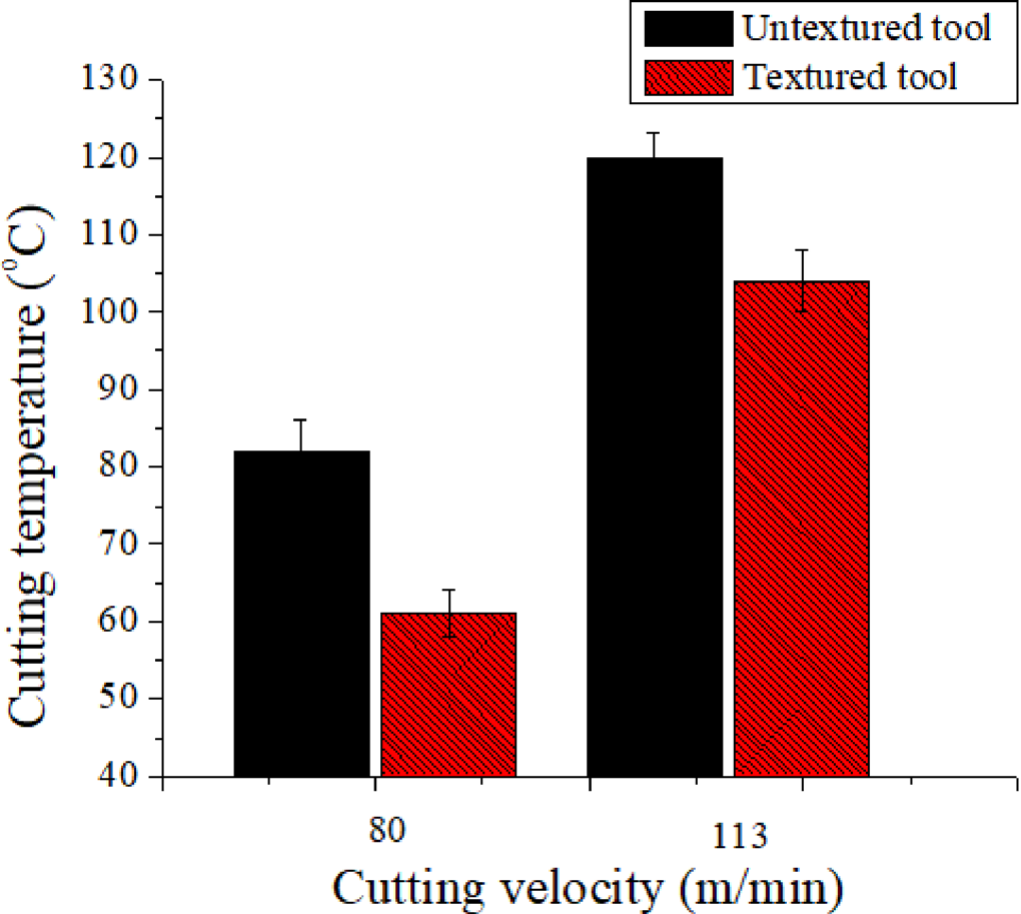
Hybrid texturing tool’s impact on cutting temperature.

**Fig. 5:**
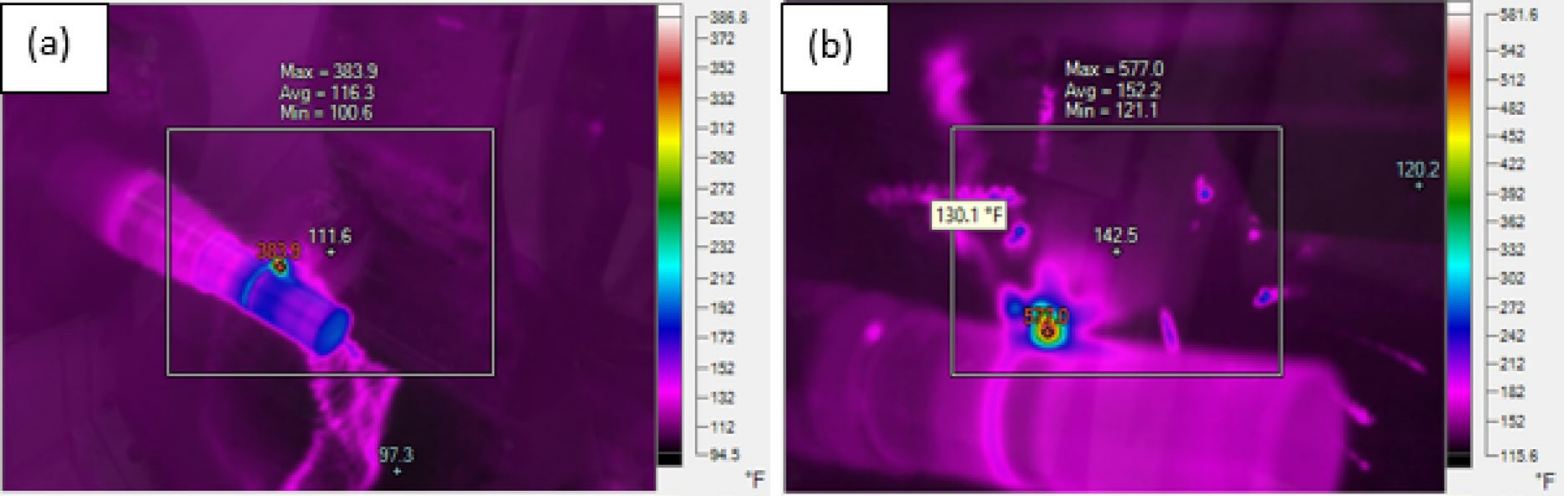
Thermal Image camera obtained sample images with untextured tool at a ‘v’ of (a) 80 m/min (b) 113 m/min.

Figure 4 depict the coolant and texture tool integrated effect on cutting temperature. It is pragmatic that cutting temperature rised when ‘v’ rised from low to high. It is evident from Fig. 5 that noticed low temperature at a ‘v’ of 80 m/min than 113 m/min due to rise of friction at the cutting zone. As the ‘T’ It is pragmatic that 61 °C and 82 °C are the recorded temperatures in textured and conventional tools at the ‘v’ of 80 m/min. In this instance, 26% drop in cutting temperature was noticed in textured tool than conventional tool. Whereas, it was 13% drop of cutting temperature at a ‘v’ of 113 m/min over conventional tool. Textured tools were shown to have much lower cutting temperatures than untextured tools at both cutting velocity conditions with wet cooling. This is due to the fact that surface texture tools store the cutting fluid in hybrid microgrooves and allow coolant to continually access the cutting zone cause low friction hence low ‘T’. Further, minimal cutting temperature reductions were seen at a’v’of 113 m/min as compared to a ‘v’ of 80 m/min in tools with texture. The poor temperature reduction at a ‘v’ of 113 m/min is caused by friction increasing as cutting velocity increases. The obtained results were well align with literature when turning steel material^3^.

### Hybrid tool and wet cooling combined impact on rake wear

**Fig. 6:**
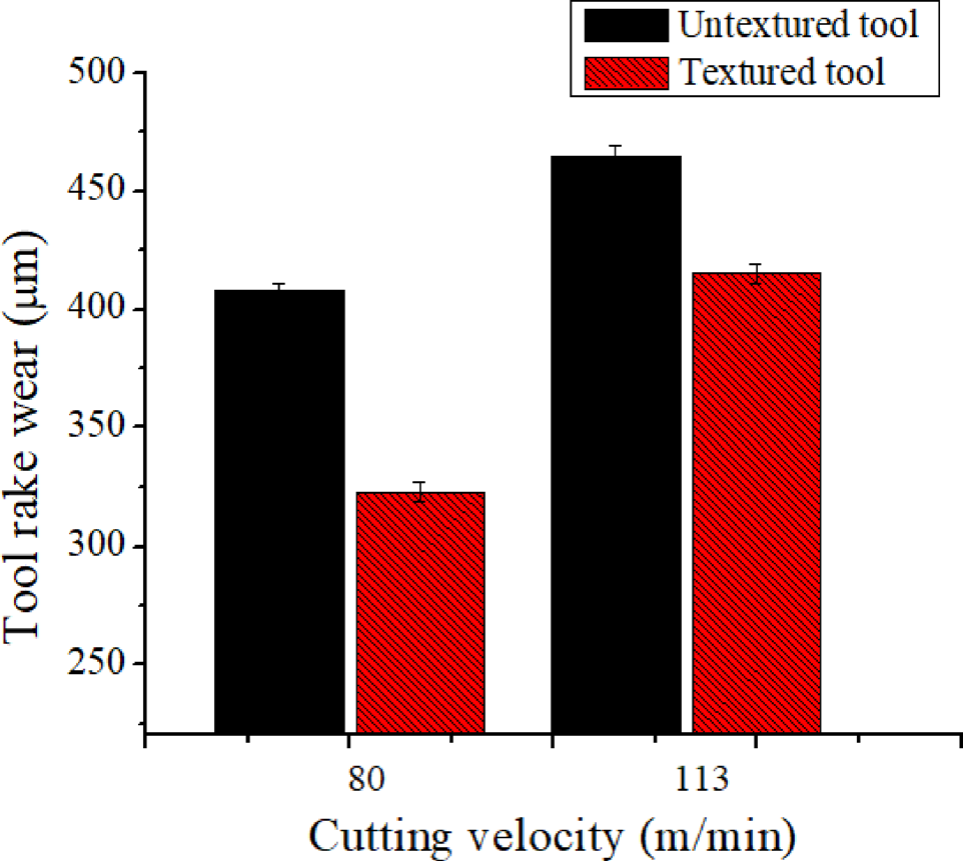
Hybrid texturing tool’s impact on ‘K_t_’.

**Fig. 7:**
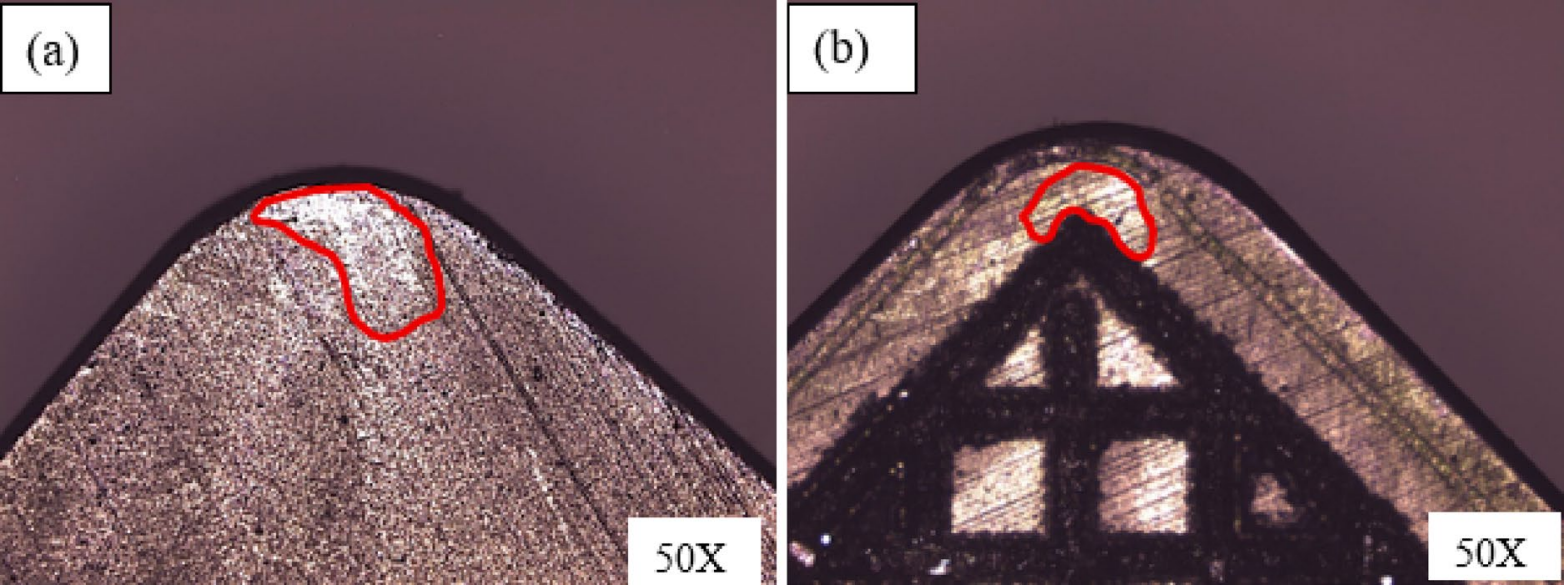
Rake wear images **a** Conventional tool, **b** Textured tool.

The effect of a textured tool on rake wear in wet conditions is shown in Fig. 6. Textured tools have less rake wear than conventional tools as shown in Fig. 6. It was pragmatic that textured tool reduced 21% and 11% rake wear at ‘v’ of 80 m/min and 113 m/min over conventional tools. This effect results from coolant storage in textured tools during wet cooling provides smooth cutting action results in low ‘L’ hence low rake wear. Another reason is that textured tool avoids chip long contact with tools due to presence of microgrooves and supports for derivative cutting mechanism. Figure 7 illustrates the attained optical microscope tool rake wear images at the given condition. Due to the chip lifting effect and derivative cutting process, shorter tool-chip contact length was seen while using textured tools, as illustrated in Fig. 7(b). Compared to conventional tools, textured tools resulted in reduced tool rake wear, as seen in Fig. 7. As seen in Fig. 7, more abrasion wear was found in untextured tool when comapred to textured tool. Further, noticed more ‘L’ in untextured tool over textured tool. Sun et al.^3^ developed different hybrid textured tool and investigated its performance during turning of AIS 1045 material and tool wear mechanism noticed was similar.

### Effect of hybrid texture tool on ‘V_b_’

**Fig. 8:**
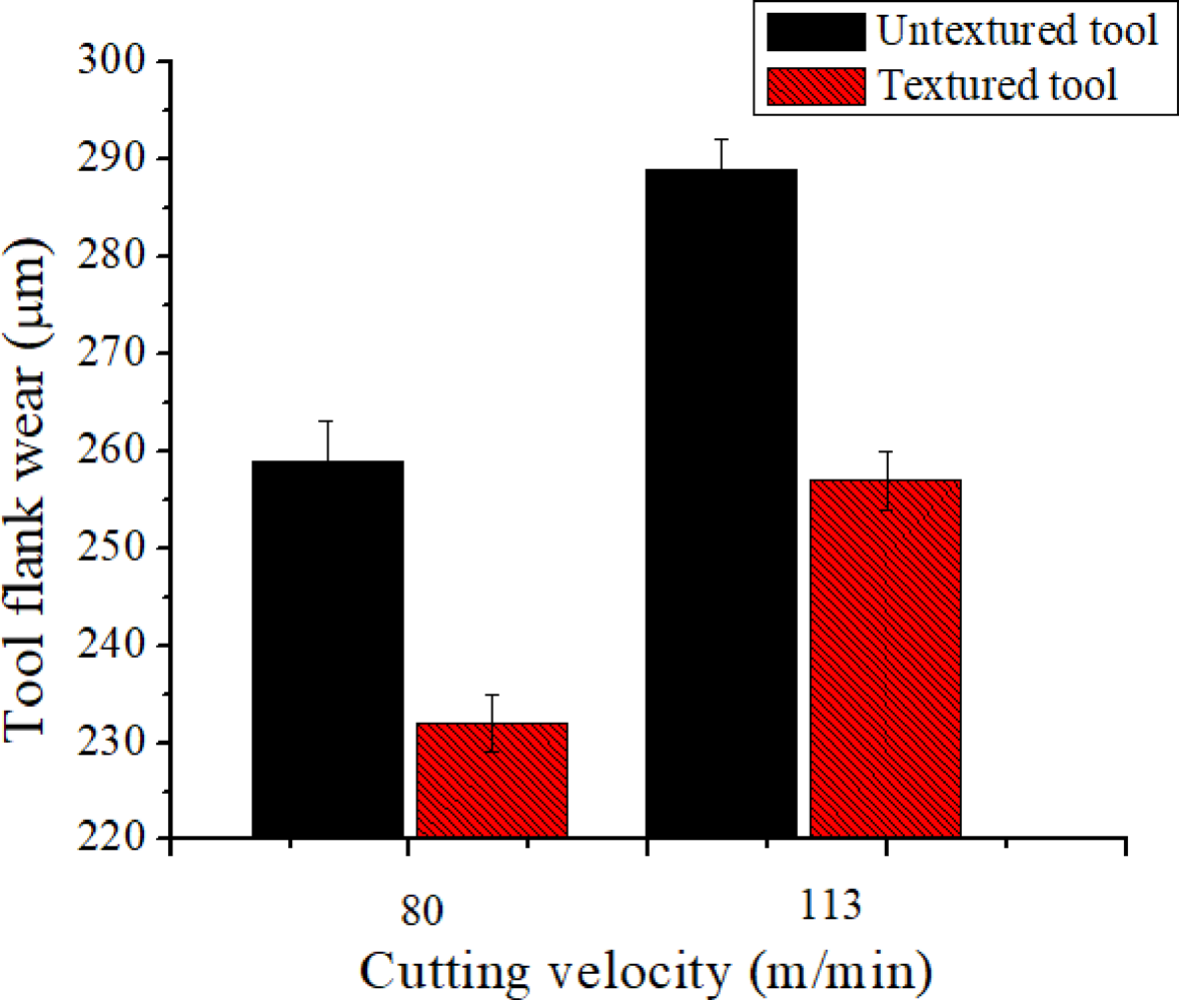
Hybrid texturing tool’s impact on ‘*V*_*b*_’.

**Fig. 9:**
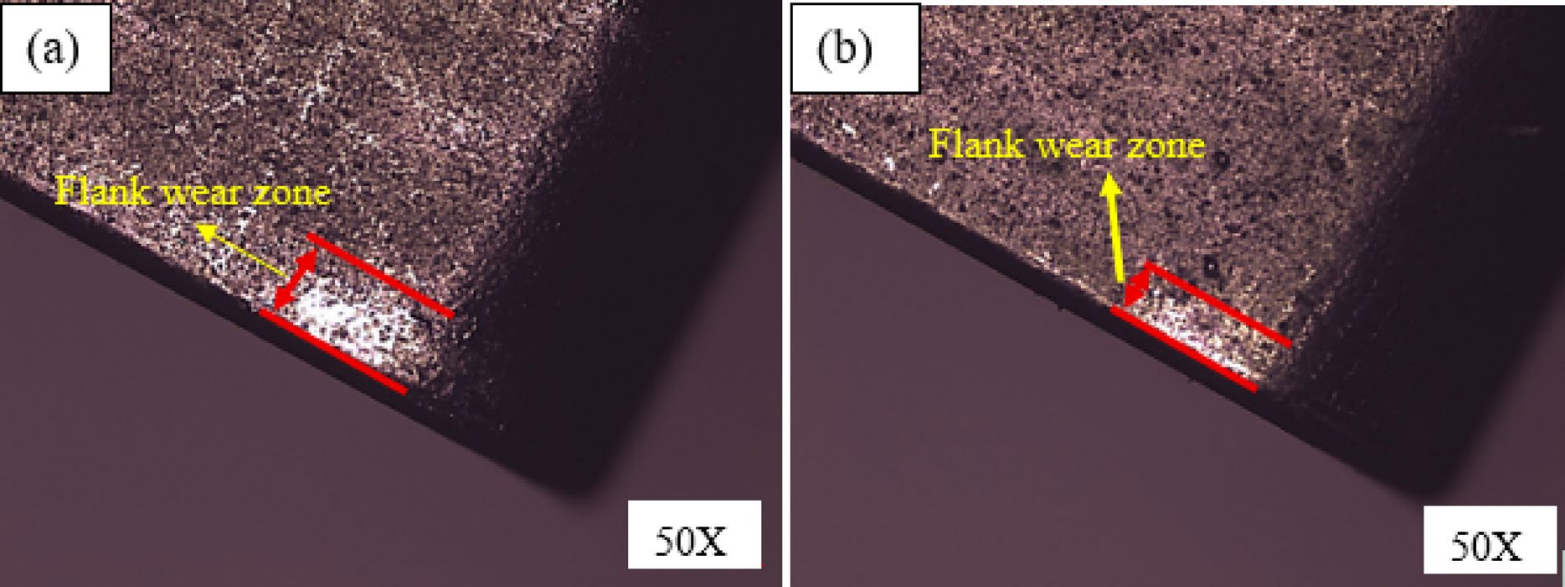
Flank wear of **a** Conventional tool **b** Textured tool.

As seen in Fig. 8, the kind of cooling method and the textured tool have a major impact on tool wear. At a ‘v’ of 113 m/min, the observed flank wear in untextured tools is 289 μm, whereas in textured tools it is 257 μm. At this stage when compared to conventional tool, 11% drop of ‘*V*_*b*_’ observed with textured. This results in effective lubrication with textured tools, causes low ‘T’ hence low flank wear. Additionally, Fig. 8 shows that the flank wear increased as cutting velocity increased. This tendency is explained by higher cutting temperatures under high cutting circumstances. It is noticed that flank was found low in textured tool over conventional tool under wet condition. Untextured tools settled with more flank wear than textured tools as depicted in Fig. 8. It noted that abrasion mechanism caused tool wear in conventional tools whereas less abrasion was noticed in textured tool as shown in Fig. 9. Further, it was observed that tool wear happened owing to abrsaion mechanaism in both textured and untextured tools. Furthermore, it was noticed maintain of cutting edge in textured tools as shown in Fig. 9(b). The findings of the current study are in good agreement with research conducted on the turning of steel^42^.

### Hybrid tool and wet cooling combined impact on ‘R_a_’

**Fig. 10:**
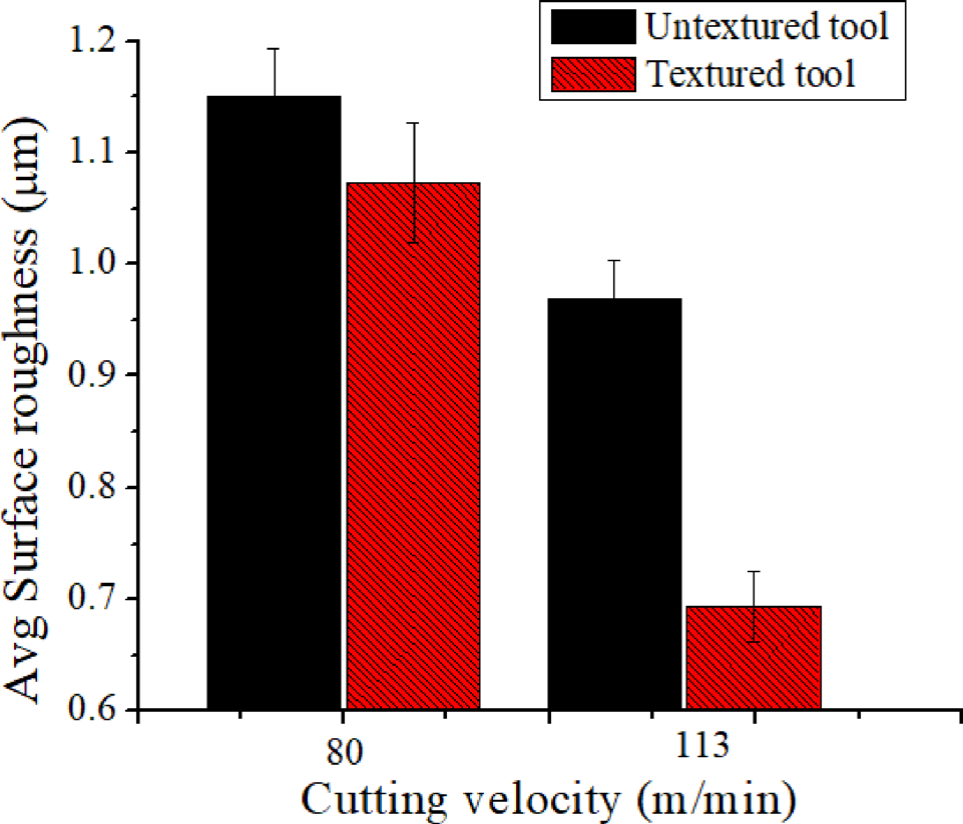
Hybrid texturing tool’s impact on on ‘R_a_’.

‘R_a_’ is one of the output responses that sensitively affect the quality as well as functional performance characteristics. Therefore, ‘R_a_’ studies were performed in the current work. Figure 10 shows that when cutting velocity rises then surface roughness followed a decreased trend. Low ‘R_a_’ is a result of less ‘L’ and cutting edge retention under conditions of high cutting velocity. According to Fig. 10, during wet cooling at both cutting velocity conditions, ‘R_a_’ found low in textured tools over textured tools. The difference is because “Ra” results from the smooth cutting action caused by lubricant retention in the textures that is efficiently fed from the microgrooves to the cutting zone. As shown in Fig. 10, the ‘R_a_’ observed values in textured and standard tools are 1.52 μm and 1.073 μm, respectively, at a ‘v’ of 80 m/min. Under these circumstances, the textured tool’s ‘Ra’ decrease was 6% more than that of the conventional tool. Additionally, at a ‘v’ of 113 m/min, a substantial 28% drop in ‘R_a_’ with textured tools. The results of the current investigation, which involved turning 17-4PH steel material with textured tools while it was cooling, closely match those found in the literature^4^.

### Hybrid tool and wet cooling combined impact on chip morphology

**Fig. 11:**
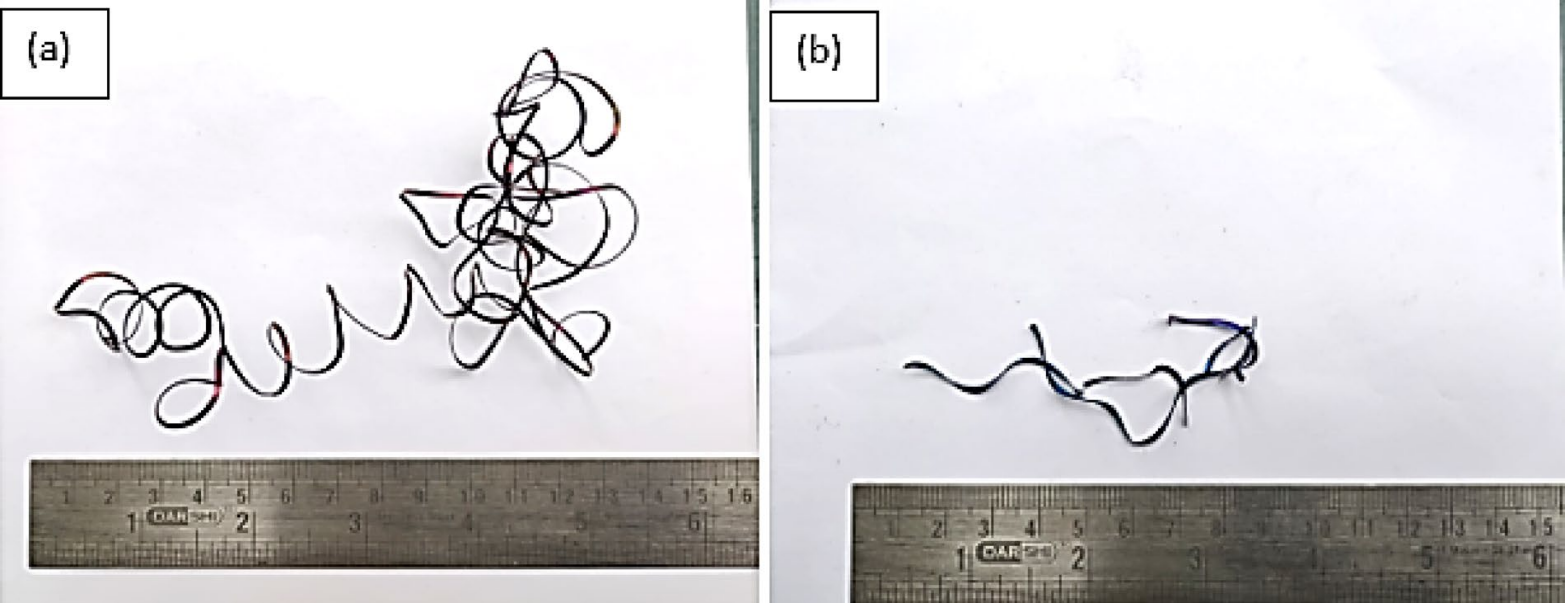
Chip morphology at a ‘v’ of 80 m/min **a** Untextured tool **b** Textured tool.

Textured tools played a significant role on type and shape of chip in the present study. Long and continuous chips were produced during cutting operation at a ‘v’ of 80 m/min in the untextured tool whereas textured tool produced discontinuous chips as shown in Fig. 11. It evident in Fig. 11 that derivative cutting mechanism cooperated in controlling the chip length in textured tool. Further, coolant stored in the microgrooves present in textured tool provides cooling effect to the chip surface at the cutting zone and supported in chip breakability more when compared to untextured tools.

### Interaction effect of process variables on output variables

**Fig. 12:**
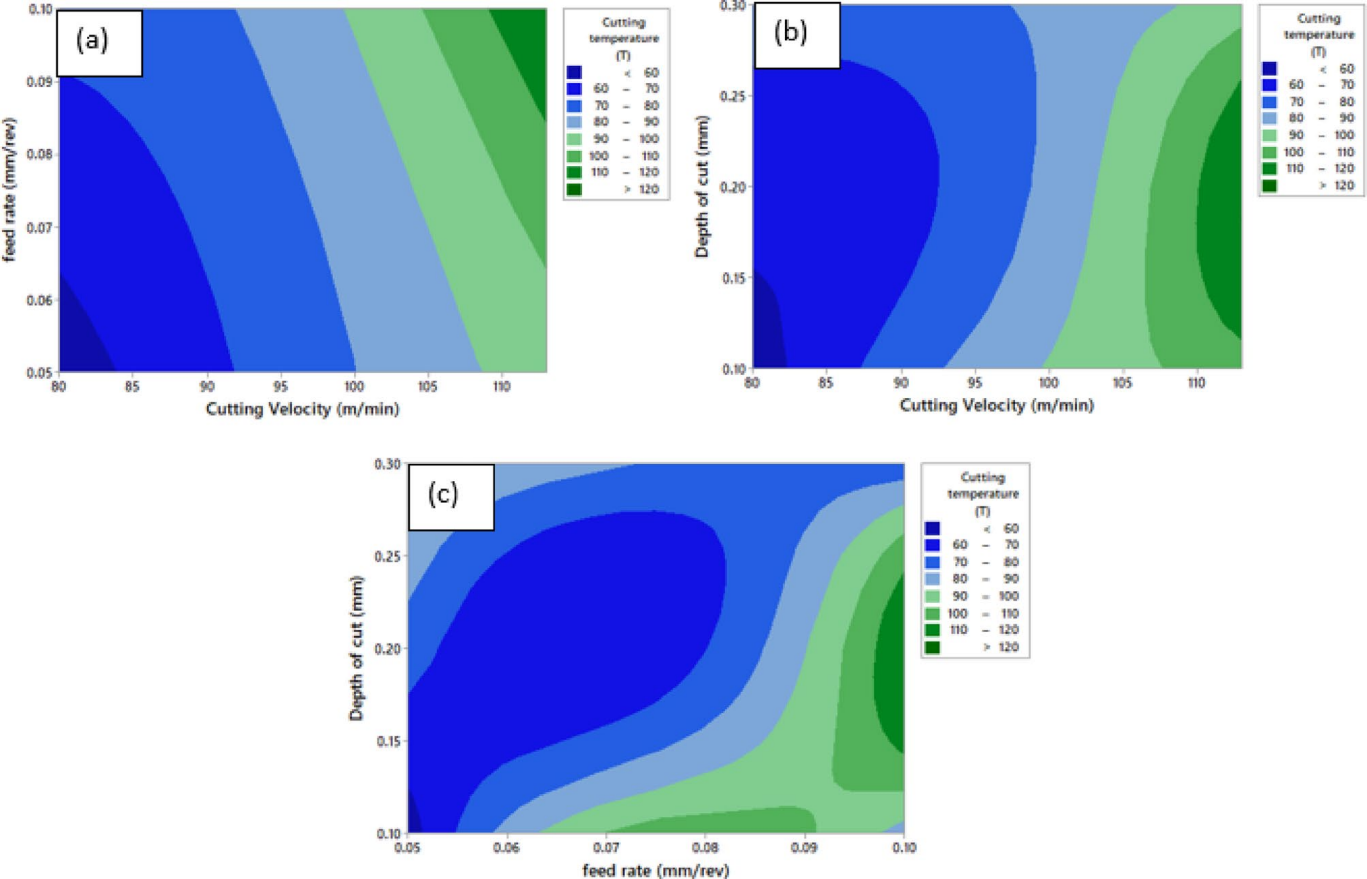
Contour Plot of ‘T’ **a** ‘T’ vs. ‘v’ and ‘f’, **b** ‘T’ vs. ‘v’ and ‘a_p_’, **c** ‘T’ vs. ‘a_p_’ and ‘f’.

Figure 12 presents contour plots illustrating the combined effects of cutting parameters on ‘T’. Figure 12(a) shows the change of ‘T’ with ‘v’ and ‘f’. It is evident that cutting velocity predominantly governs temperature evolution. At lower velocities (≈ 80–90 m/min), the temperature remains low regardless of feed rate. As velocity increases, temperature rises steadily, with the highest values (> 120 °C) observed above ≈ 105 m/min. Feed rate has a comparatively moderate effect, producing a slight temperature increase at higher velocities, likely due to enhanced frictional interactions. Figure 12(b) depicts the interaction between ‘v’ and ‘a_p_’. Consistent with the previous observation, increasing cutting velocity significantly elevates temperature. At shallow depths, temperature remains low at small velocities, whereas higher velocities combined with greater depths of cut expand high-temperature zones. This is due to larger ‘L’ and increased cutting forces, which intensify heat generation. Figure 12(c) illustrates the combined effect of ‘a_p_’ and ‘f’. The contours indicate that temperature rises with simultaneous increases in both parameters. Low feed rates and shallow depths maintain minimal temperatures, while higher values of both lead to elevated temperatures due to increased chip load, plastic deformation, and friction at the cutting zone. Overall, it was observed from contour plots that ‘T’ significantly affected by the ‘v’.

**Fig. 13:**
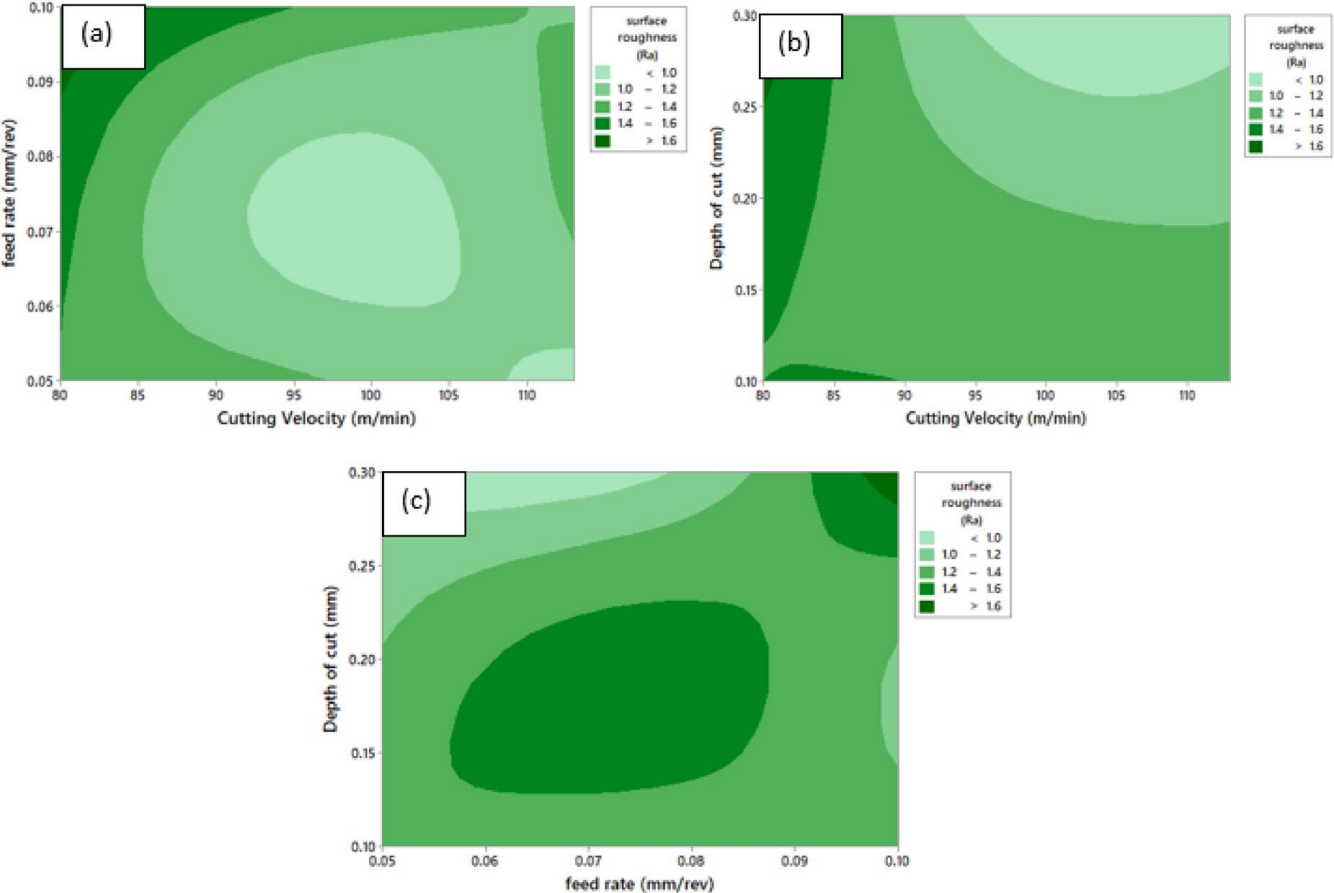
Contour Plot of ‘R_a_’ **a** ‘R_a_’ vs. ‘v’ and ‘f’, **b** ‘R_a_’ vs. ‘v’ and ‘a_p_’, **c** ‘R_a_’ vs. ‘a_p_’ and ‘f’.

Contour graphs showing the combined impact of machining variables on ‘R_a_’ are shown in Fig. 13. Figure 13 (a) shows the interaction between ‘v’ and ‘f’, ‘R_a_’ is predominantly governed by feed rate over the entire range of cutting velocities considered. Lower Surface roughness values are attained at moderate ‘v’ (≈ 90–105 m/min) in conjunction with low to moderate ‘f’. An increase in ‘f’ leads to higher ‘R_a_’ regardless of ‘v’, owed to the upsurge in uncut chip thickness and consequent degradation of surface quality. Relatively higher Surface roughness at very low cutting velocities are obtained due to unstable cutting conditions and enhanced cutting temperatures. Figure 13(b) illustrates the combined influence of ‘v’ and ‘a_p_’. The contours reveal that cutting velocity exerts a stronger influence on surface roughness than ‘a_p_’. Reduced ‘R_a_’ is observed at higher cutting velocities, particularly at shallow to moderate depths of cut. In contrast, lower cutting velocities result in a marked increase in ‘R_a_’, especially at higher depths of cut, owing to intensified tool–workpiece interaction. Overall, the effect of ‘a_p_’on ‘R_a_’ remains comparatively limited within the examined range. Figure 13(c) depicts the interaction between ‘a_p_’ and ‘f’. The‘f’ emerges as the most dominant factor affecting ‘R_a_’, with ‘R_a_’ increased substantially as ‘f’ increases, particularly at moderate to higher depths of cut. The minimum roughness region occurs at low ‘f’ and shallow ‘a_p_’, while higher ‘R_a_’ values persist at elevated feed rates irrespective of depth of cut. This trend is mainly associated with pronounced feed marks and higher material removal per revolution. Overall, the contour plots confirm that ‘f’ has the most pronounced effect on ‘R_a_’, followed by ‘v’. The optimal surface finish is achieved at ‘f’ combined with moderate to high ‘v’and lower ‘a_p_’as depicted in Fig. 13.

**Fig. 14:**
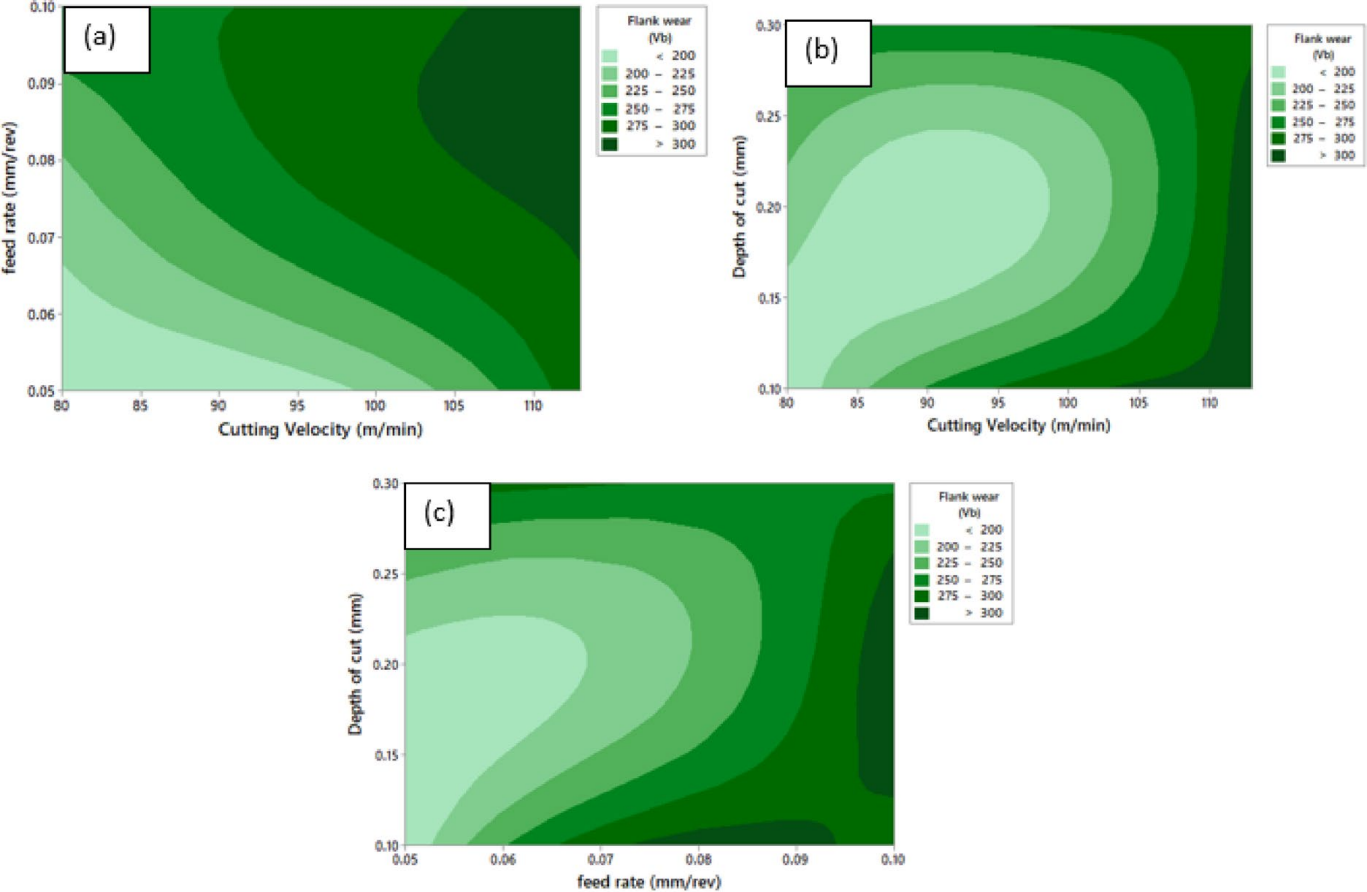
Contour Plot of ‘V_b_’ **a** ‘V_b_’ vs. ‘v’ and ‘f’, **b** ‘V_b_’ vs. ‘v’ and ‘a_p_’ , **c** ‘V_b_’ vs. ‘a_p_’ and ‘f’.

Machining parameters influence on ‘V_b_’ is shown in Fig. 14. Figure 14(a) shows the combined effect of ‘v’ and ‘f’ on flank wear, revealing a clear increasing trend with the simultaneous increase of both parameters. Lower flank wear is obtained at reduced ‘v’ and ‘f’, indicating diminished thermal and mechanical loading on the cutting edge. An increase in ‘f’ results in a pronounced rise in ‘V_b_’ across the ‘v’ range, primarily due to higher chip load and intensified abrasive interaction at the cutting interface. Likewise, higher cutting velocities accelerate wear as a consequence of elevated ‘T’. Figure 14(b) presents the interaction between ‘v’ and ‘a_p_’. The contours indicate a noteworthy increase in ‘V_b_’ with increasing ‘v’, particularly at higher depths of cut. A distinct minimum wear region is observed at low cutting velocities combined with shallow depths of cut, where cutting forces and heat generation remain relatively low. Conversely, the combination of higher depths of cut and elevated cutting velocities leads to severe flank wear, attributable to increased contact stresses, higher material removal rates, and greater thermal softening of the tool material. Figure 14(c) demonstrates the combined effect of ‘a_p_’ and ‘f’ on flank wear. The results show a progressive upsurge in ‘V_b_’ with increases in both parameters, with feed rate exhibiting a marginally stronger influence. Minimum flank wear occurs at low ‘f’ and shallow ‘a_p_’. At higher ‘f’, ‘V_b_’ increases substantially regardless of ‘a_p_’, indicating the dominance of abrasive and adhesive wear mechanisms associated with greater chip thickness and prolonged tool–workpiece contact. Overall, the contour plots confirm that ‘a_p_’ is significantly pretentious by ‘v’ and ‘f’ while ‘a_p_’plays a secondary role. Conditions conducive to minimizing flank wear are achieved at lower ‘v’, ‘f’ and ‘a_p_’ as evidenced in Fig. 14.

**Fig. 15:**
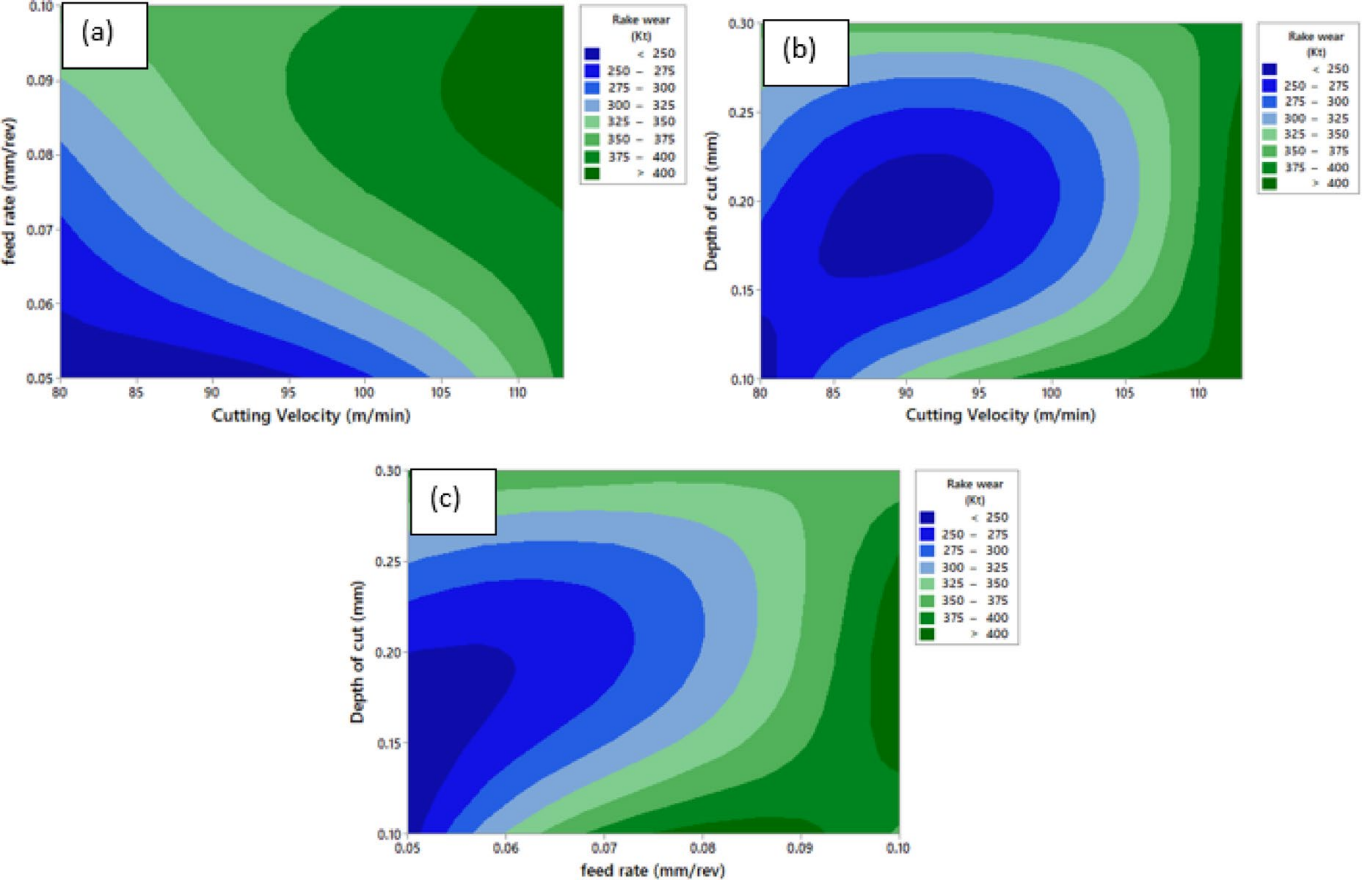
Contour Plot of ‘K_t_’ **a** ‘K_t_’ vs. ‘v’ and ‘f’, **b** ‘K_t_’ vs. ‘v’ and ‘a_p_’, **c** ‘K_t_’ vs. ‘a_p_’ and ‘f’.

Figure 15 illustrates contour plots representing the combined influence of machining parameters on K_t_. The colour gradient represents the variation in rake wear, with darker blue regions indicating lower wear and green regions corresponding to higher wear values. Figure 15(a) shows the interaction between ‘v’ and ‘f’. An overall increase in rake wear is observed with increasing ‘v’ and ‘f’. Lower rake wear values are achieved at lower ‘v’ and ‘f’, where the thermal and mechanical stresses acting on the rake face remain relatively low. As feed rate increases, rake wear rises consistently over the entire cutting velocity range due to increased chip thickness and intensified tool–chip interaction. In addition, higher cutting velocities promote accelerated rake wear because of elevated temperatures that favor diffusion- and adhesion-driven wear mechanisms. Figure 15(b) presents the combined effect of ‘v’ and ‘a_p_’ on rake wear. The contour patterns indicate that ‘K_t_’ increases with ‘v’, particularly at moderate to higher depths of cut. A clear low-wear region is observed at lower cutting velocities combined with shallow depths of cut. Conversely, higher cutting velocities in conjunction with increased depths of cut result in severe rake wear, mainly due to an enlarged contact area on the rake face, higher material removal rates, and increased thermal loading. Figure 15(c) illustrates the interaction between ‘a_p_’ and ‘f’. ‘K_t_’ is found to increase progressively with both parameters, with feed rate having a more pronounced effect. Minimum wear occurs at low ‘f’ and shallow ‘a_p_’. At elevated feed rates, rake wear increases significantly irrespective of depth of cut, highlighting the predominance of adhesion and diffusion wear mechanisms associated with increased chip–tool contact and longer interaction duration. Overall, the contour plots demonstrate that ‘v’ and ‘f’ emerging as the most influential factors on ‘K_t_’. Conditions favouring lower rake wear correspond to lower ‘v’, ‘f’ and ‘a_p_’ as evidenced in Fig. 15.

## Conclusions

The present work used developed hybrid tools and performance machinability studies in the turning of 17-4PH steel material. From results, observed the below points.

- Textured tools allowed coolant storage in the continuous microgrooves resulting in low T, R_a_, V_b_ over conventional tools.
- Textured tools exhibited significantly lower ‘T’ than conventional tools under wet cooling at both cutting velocities, achieving a reduction of approximately 26% at 80 m/min and 13% at 113 m/min.
- The effectiveness of tool texturing decreased at higher ‘v’ due to increased friction and limited coolant penetration.
- An increase in ‘v’ led to higher ‘V_b_’ due to increased thermal and mechanical loading, while textured tools maintained reduced wear and improved edge stability.
- The primary tool wear mechanism in both tools was discovered to be abrasion only.
- Textured tools achieve lower ‘R_a_’ than conventional tools, showing reductions of 6% at 80 m/min and 28% at 113 m/min, due to enhanced lubricant delivery from the microgrooves.
- At the high cutting velocity condition, both textured and untextured tools produced surfaces with low surface roughness.
- Surface texturing modifies the tool–chip interaction, resulting in segmented chips and improved control over chip length.
- Overall, the contour plots demonstrate that both tool wears and cutting temperature are strongly affected by cutting velocity whereas it was feed rate in the case of surface roughness.
- Metal cutting industries could employ the developed tools to enhance the machinability of 17-4PH steel.

### Future scope

Future work should explore hybrid textured tools under a wider range of cutting conditions, investigate integration with advanced cooling strategies, and optimize texture geometry to further improve wear resistance and surface quality.

## Data Availability

The datasets generated during and/or analyzed during the current study are available from the corresponding author on reasonable request.
